# Contact Observations from an Intensive Care Unit

**DOI:** 10.1038/s41597-025-05249-5

**Published:** 2025-06-04

**Authors:** Hieu Vu, Roger Struble, Philip M. Polgreen, Bijaya Adhikari, Ted Herman

**Affiliations:** 1 Department of Computer Science, University of Iowa, Iowa City, US; 2 Department of Internal Medicine, University of Iowa, Iowa City, US

**Keywords:** Epidemiology, Health services

## Abstract

Location and interaction data for workers in a hospital unit are useful for epidemiological research. This article describes a one-week study measuring contacts between healthcare professionals in a medical intensive care unit. Measurements capture the duration of contact, defined as being within six feet (1.8 ± 0.1 meters) distance between instrumented persons or between persons and selected locations throughout the unit. Within each patient room, measurements distinguish between three places: the sink, the computer, and the vitals monitor. Data from the study, approximately 15 million records, are processed into different formats that facilitate analysis.

## Background & Summary

Data on the dynamics of infectious diseases are critical in combating future epidemic outbreaks. Based on such data, mathematical or simulated models can be constructed, which contribute to understanding how pathogens spread and enable hypothetical testing of interventions. The accuracy of the model is grounded in the quality of empirical data collected from the real world, which informs the model parameters. For example, mechanistic models of the COVID-19 outbreak may include parameters such as probability and duration of contact^1^, the basic reproduction number^2^, recovery and mortality rate^3^, and the asymptomatic infectivity rate^4^, all of which had to be estimated from empirical contact tracking data^5^ and mobility data^6,7^. Similarly, models on spatiotemporal dynamics^8,9^ and heterogeneous agents^10^ rely on mobility data to estimate essential parameters. The probability of disease transmission can depend on the duration of exposure to an infected person or pathogen.

Healthcare-Associated Infections (HAIs), which primarily spread in hospitals and clinics, lead to increased morbidity and mortality and pose significant financial strains^11,12^. HAIs have been investigated in various ways: to estimate risk factors^13,14^, to model transmissions^15^, to detect outbreaks^16,17^, and to design intervention strategies^18,19^. These investigations have revealed insights into the spread of HAI, albeit often relying on in-silico experiments conducted over synthetic data. Such studies could benefit from more empirical data provided by healthcare facility measurements. While patient-to-patient interaction is expected to be minimal within healthcare facilities, contacts between Healthcare Professionals (HCPs) and patients during care delivery are possible pathways for HAI transmission. Contacts between HCPs and contacts with medical equipment could be indirect transmission routes for HAIs. Measurements of HCP movement, dwell time, and various contacts add value to models of HAI transmission.

Previous research, with similar motivations to the study reported here, uses wireless sensors to record HCP movement and potential contacts in hospital^20–33^. Results in obtaining data vary in accuracy of measurement (which depends on the technology of the sensors); in duration and scope (with sampling considerations); spatial and temporal granularity of measurement. One feature of the data reported in this paper is the sensing technology, which is based on Ultra Wide-Band (UWB) devices to measure proximity. UWB has better accuracy than comparable wireless devices: reliable ten-centimeter accuracy in distance measurement. UWB has been used in at least one medical application: to monitor hand hygiene^34^. Our work applies UWB to the task of measuring contacts among HCPs and between HCPs and selected locations or objects. The data distinguishes several specific areas within each patient room of the Medical Intensive Care Unit (MICU).

The Methods Section explains the operation of the study, the notion of a contact interval, UWB badges, study limitations, and methods of converting raw data into more convenient forms described in the Data Records Section. The study data contains records of contacts between HCPs, relevant to network analysis, and records of contacts between an HCP and instrumented locations in the MICU, relevant to tracking movement.

## Methods

### Quick Overview

Initial data is put into two other forms: contact intervals and imputed history, which are for convenient data analysis. In addition to the data, some files provide location information and the layout of the MICU. The initial data consists of records for each badge worn in the MICU that either represent the initiation of a contact or the termination of a contact. Later in this section, we explain the notion of a contact interval, and we discuss some design rationale for the study, constraints, and limitations. From the contact intervals, an imputed history of badge contacts with one-second resolution is derived. The imputed history makes it easier to answer queries such as: how much time healthcare providers spend in patient rooms and what the average transit time is for nurses who move directly from one patient room to another patient room. Using the imputed history, it is simple to construct a contact graph for a specific second *t* or for a period of time, such as an hour [*t*, *t* + 3600].

### UWB Badges for Contact Detection

One refinement over previous work on measuring HCP contacts is our goal to measure distinct locations within patient rooms, in addition to recording contacts between HCPs. This is possible due to characteristics of UWB badges^34^, which detect distance with an accuracy of ten centimeters; this allows us to denote contact distance as 1.8 ± 0.1 meters. Previous comparable studies^23–33^ have used radio (e.g., active RFID) where a receiver obtains an RSSI (Received Signal Strength Indicator) value for a message received. RSSI is susceptible to noise caused by multipath effects^35–38^. While there are techniques to mitigate this noise, e.g., by sampling at multiple radio frequencies, the resulting distance calculation does not reach the accuracy of UWB.

The COVID-19 pandemic (2019–2022) motivated intense efforts to track and monitor the spread of the disease. One commercial product devised for the pandemic, called Instant-Trace, was a system based on UWB badges, a supporting smartphone app, and cloud-based administration with data storage. It is useful to understand the vision of Instant-Trace and how we used the system to obtain the data presented in this paper. Briefly put, the design of Instant-Trace resembles monitoring exposure to radiation in an industrial workplace where each worker wears a badge that measures cumulative radiation exposure; workers check out at the end of each shift at a kiosk, which offloads data from the badge, uploading these data to a dashboard that managers inspect; the kiosk scan also clears badge memory to prepare for future shift data. Instead of radiation, the Instant-Trace system tracks cumulative exposure time, where exposure is defined as being within six feet (1.8 meters) of other badge-wearing workers. This distance setting was motivated by the national agency, U.S. Centers for Disease Control and Prevention (CDC), which recommended six feet for social distancing during the pandemic^39^. The Instant-Trace system neither depends on infrastructure (e.g., WiFi, cellular, etc.) nor modifications to the workplace. Tracking exposure is achieved by exchanging messages directly between worn badges.

### Deficiencies of Badges

Badges do not continuously sense other badges; instead, they use a periodic radio signaling cycle to gather responses from badges in range and determine which are within a predefined contact distance threshold. When two badges are within the distance threshold, a *contact* between them is recorded. The frequency of this low-level signaling cycle is on the order of a few seconds for reasons of efficiency and battery life conservation. Badges do not, therefore, instantly sense contacts and could even miss detecting contacts (e.g., two badge wearers at high speed are briefly under the distance threshold but are missed by the repeated signaling cycle).

Badges have limited capacity for data. At a higher level in the badge operating system, with a lower frequency (once every 15 seconds), the processor monitors ongoing contacts with other badges, deciding when to consider a new contact or purge some current contacts. It is this higher-level cycle that adds to an in-memory list of badge *contact intervals*: a contact interval is intended to be a period during which two badges are continuously within the distance threshold. The Instant-Trace architecture, with its low-level signaling and higher-level processing cycles, results in imperfect contact interval implementation: very brief contact intervals may be missed, and the start or end times of contact intervals are approximate. Furthermore, badges do not have high-precision or synchronized clocks.

Although the choice of processor, the algorithms for sensing, recording, and offloading of contact data are artifacts of how the Instant-Trace badge system was designed, there are fundamental limits of any badge system: cost, memory, frequency of sampling, battery lifetime, infrastructure dependency, and weight (packaging) are elements of design trade-offs. We acknowledge that the results and limitations of this study may be improved with other UWB instruments.

### Badge Specialization

The badges have a feature to conserve battery power: if a badge is not being worn, it enters sleep mode with contact sensing turned off. For our study, badges were used in two ways, either worn by an HCP or put in a stationary location; the latter we name *anchors*. The battery conservation feature was disabled during the study so that the contact distance between an HCP and an anchor was reliably measured and recorded.

Within the MICU, patient rooms are of special interest in epidemiology: patients in an ICU can have weakened or compromised immune systems; a patient room can be a nexus of infection spread. The patient rooms differ in size and layout. Four anchors were placed in each patient room: at the door to the room, near the sink, at the computer workstation, and on the vitals monitor. The vitals monitor is close to the patient’s bed and thus a proxy for possible physical contact with the patient. Care was taken so that no two anchors (throughout the MICU) were within six feet of each other to avoid generating anchor-to-anchor contacts.

**Fig. 1:**
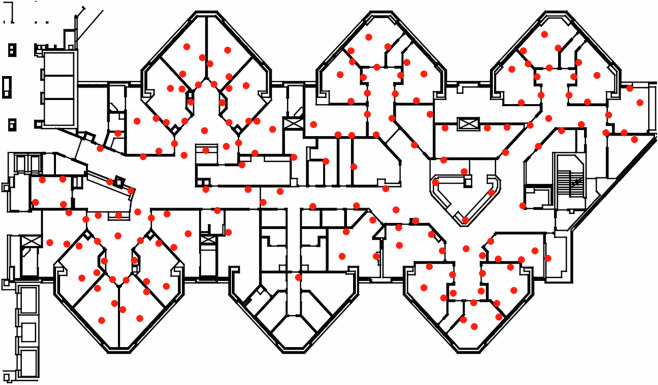
MICU Layout With Anchor Placement. The center bottom pod of rooms has no anchors: this is an office area with no patients and outside of HCP activity.

Figure 1 shows the locations of anchors, represented by red dots, in the layout of the MICU. Each badge has a unique internal identifier, which the data encodes as a short label that indicates the role of the badge: examples are b037 for anchor 37, pr015 for an HCP in a provider capacity, n008 for a nurse, and ss044 for a support-staff HCP (dietician, therapist, etc.).

### IRB and Operation of the Study

University of Iowa Institutional Review Board (IRB# 201904806) approved the conduct and sharing of the data. No patient data was collected for this study. Moreover, there is no indication in the data of whether or not a patient room is occupied; usually, the MICU is fully occupied. The IRB approved staff to wear badges subject to the constraint that no individual could be identified from the data collected. HCPs were neither compelled to wear nor compensated for wearing badges during the study. All HCPs tasked to work in the MICU did wear badges for this study. The IRB approved a waiver of documentation of consent because the study presents no more than minimal risk, and the only data being collected from participants were job location and job type. Accepting and wearing a badge indicated their consent to participate.

The data is derived from a one-week study of the University of Iowa main hospital’s Medical Intensive Care Unit (MICU), which has 26 (single-occupancy) patient rooms. The study’s main idea was to have all HCPs wear badges that measure their proximity to each other and to selected locations or objects in the MICU. Each day’s work in the MICU is divided into two 12-hour shifts, the day shift and the night shift. Our team arranged for badges to be given to each HCP at the beginning of a shift and collected (handed back in) for data extraction when the HCP finished the shift.

The four categories of badges used in the study consisted of nurses, providers (physician roles), support staff (nutritionists, therapists, etc.), and anchors. We overprovisioned the number of badges for each HCP category and randomly selected a badge for MICU staff when they began their shifts. Badges were reused during the study (the same badge worn by different persons), but not during any single shift; thus, the integrity of any badge data for analysis (e.g., a contact graph) is valid per shift. Unlike the badge vendor’s intended data extraction from badges via a kiosk, we used the vendor’s smartphone app to scan badges. Members of our team also used this app to scan anchors twice daily (not doing so would have exceeded an anchor’s memory capacity for contact data).

The MICU is unlike many other hospital wards: patients are rarely ambulatory, the ratio of nurses to patients is high, and for some patients, there are extra precaution measures (gowning and masks). For day shifts, which run from 07:00 to 19:00, the roster numbers are: 12 providers (physicians of different types), 19 nurses, and 12 support staff (therapists, nursing assistants, clerks, pharmacists, and some medical students); the respective numbers for the night shift are 6, 15, and 8. Sometimes staff are assigned temporarily to other units of the hospital, hence, these numbers can vary. The data for each shift typically contains more badge identifiers than one would expect from roster numbers for a single shift; the Study Limitations subsection discusses this in more detail. The MICU operates as a *closed model* intensive care unit^40^: consultant providers, who we expect visited their patients at most once per day, did not wear badges. Non-staff visitors and patients did not wear badges. No social distancing policy was in effect during the study; many HCP contacts within six feet are visible in the data.

### From Raw Data to Contact Intervals

This subsection explains the process of creating contact intervals from raw data. Data extracted from a badge is a sequence of records with fixed fields. These records are of two types: either observing the start of a contact interval or the end of a contact interval. Our construction of contact intervals takes care of timing imperfections and brief contacts.

1. The program that extracts records from the vendor’s database gathers all badge observations, not necessarily in order. After sorting by the time of initial contact, overlapping contact intervals are visible: for example, a nurse could be simultaneously in contact with another nurse and a provider (physician).
2. The Instant-Trace system attempts to synchronize the clocks of all badges when badges are scanned. Their clocks can drift during the time between the initial badge handout and check-in at the end of a shift. In theory, each contact interval between a pair of badges, say *a* and *b*, should be represented by records of *a* and records of *b* with the same time of observation. In practice, we found a time discrepancy of some seconds. A further consideration is the lag between physically moving to within a six-foot distance and the detection thereof. That is, even if *a* and *b* had perfect clock synchronization, it could be that *a* detects (*a*, *b*) contact a few seconds earlier than *b* detects (*b*, *a*) contact because their periodic sensing cycles may not be synchronized.
3. The combined sequence reveals that the expected pattern of a contact interval is sometimes broken: there can be a record that starts a contact interval, yet no subsequent record ends the interval. This occurrence reflects an Instant-Trace design choice: the system effectively ignores contacts in a period of less than 15 seconds. For the purposes of our study, the presence of such broken intervals is useful for geographic contacts. Our approach for these incomplete intervals is to estimate a contact interval of 10 seconds.

#### Construction of Contact Intervals

**Fig. 2:**
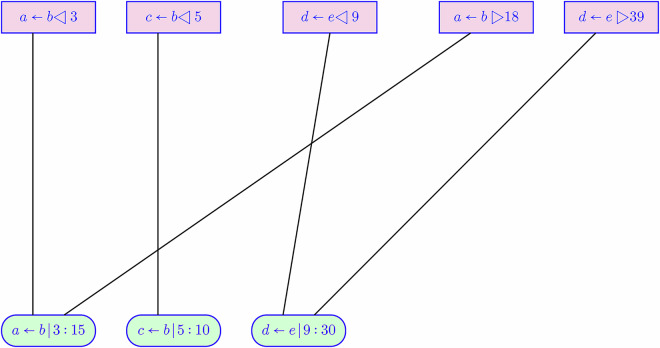
Illustrating Raw Data Collation. The top row represents raw data records, with *p* ← *q*◁ *k* denoting reporting badge *p* detected the start of a contact interval with badge *q* at time *k*; *p* ← *q* ▷*k* is the end of the contact interval. The bottom row shows derived contact intervals, with *p* ← *q*|*k*: *m* representing a contact interval between *p* and *q*, starting at time *k*, having duration *m* seconds. For contact intervals derived from matching start and end records, *m* will be a multiple of 15; for contact intervals lacking an end record in the raw data, the substitute value for *m* is 10.

Figure 2 shows a simplified view of how raw data records are collated into the sequence of contact intervals. The method of combining overlapping contact intervals is a union operation. The union works as follows: each interval for a pair (*a*, *b*) that overlaps in time with an interval (*b*, *a*) rewrites both intervals to have the same duration based on the minimum start-of-contact and the maximum calculated end-of-contact times. This rewriting is motivated by the fact that clocks within badges can drift apart by some seconds (they are synchronized with each download scan at the start or end of a shift). Figure 2 does not show all intervals. For example, the presence of *a* ← *b* |3: 15 implies that *b* ← *a* |3: 15 will also be present in the data for contact intervals.

The Data Records section presents the format of raw data and contact interval records. One field in each record is a distance value, reported in inches, obtained by UWB sensing around the time of the contact interval. We observed cases where this reported distance was only a few inches, which is an unlikely value for HCPs wearing badges in the MICU; such short distances occur when badges are powered on and together (e.g., in a receptacle) at the end of shift when turned in, or before handout to an HCP. To handle such cases, we skip over raw data records with a sensed distance of less than 12 inches.

### From Contact Intervals to Imputed Badge Histories

An alternative representation of data would be a history ${\mathcal{H}}$, indexed by time, of each HCP’s status, such as their location and which other HCPs are in contact. We are unable to achieve this exact representation from our data: there are times for which an HCP may be out of contact range from any badge or anchor in the MICU (because coverage of the MICU is limited). However, we can approximate ${\mathcal{H}}$ from contact intervals; this approximation is an *imputed history*.

**Fig. 3:**
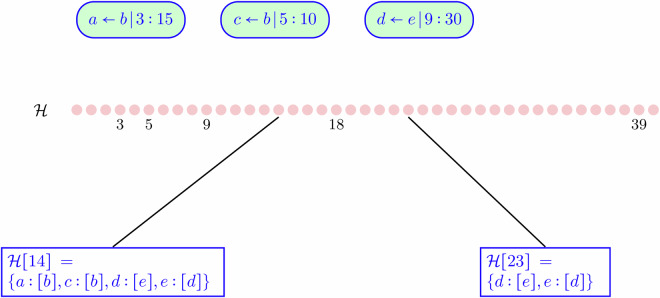
From Intervals to Contact History. Here we assume *b* is an anchor and look at two times, 14 and 23. ${\mathcal{H}}[14][b]$ is not present because anchors are omitted as primary entries in history; however both ${\mathcal{H}}[14][a]$ and ${\mathcal{H}}[14][c]$ return the list [*b*], indicating that both *a* and *c* are in contact with *b*. Had *a* also been in contact with another badge *k* at time 14, then ${\mathcal{H}}[14][a]$ would be [*b*, *k*]. At time 23, only the contact between *d* and *e* remains, represented in both directions.

The method is to first construct ${\mathcal{H}}$ to be a map from each second *t* to an empty list. Then, by iterating over values of *t*, we search contact intervals to find the set ${{\mathcal{C}}}_{t}$ containing intervals for which *t* is between the start and end of the interval. Because our goal is to track HCPs, we omit the status of anchors in this procedure, although information about anchors indirectly remains, thanks to the symmetry of contacts. Figure 3 illustrates how this works, using a simplified representation. Construction of ${\mathcal{H}}$ merges bidirectional contacts (in fact, forces this symmetry) so that if an HCP badge *d* reports contact with an HCP badge *e*, then *e* will also indicate contact with *d* at the same time.

Each history ${\mathcal{H}}$ spans one shift, justified by the following considerations. Recall that the operation of the deployment began each shift with a random assignment of badges to HCPs. Indeed, over the course of the fourteen 12-hour shifts, some badges were used several times by different HCPs; no such badge reuse occurred within a single shift. Thus, the validity of badge identity within any particular shift history ${\mathcal{H}}$ is preserved. Each node of a contact graph for a shift represents one healthcare professional.

### Geographic Enhancement to Imputed History

The set of histories enables the construction of event and motion replays for each shift (e.g., to make a sequence of contact graphs for an hour in the MICU). We anticipate questions guided by geographic considerations, particularly time spent by HCPs in patient rooms, which are critical areas for infection control. Knowing whether or not, with respect to a given history ${\mathcal{H}}$, a particular HCP is, or is not, in a patient room may not be an elementary question. A heuristic for in-room inference is described below; it is one example showing how an inference may enrich an imputed history.

#### Door vs. In-Room

When is an HCP detected to be in a patient room? Sensing contact with the door anchor is insufficient evidence. It could be that an HCP lingers at the door, but does not enter the room. Alternatively, it could be that the HCP passes through the doorway (which is detected as a contact), then dwells for a time in the room, but does not contact any other anchor within the room. A conservative, more specific answer occurs when the HCP has contact with one of the interior anchors (sink, computer workstation, or vitals monitor): such contact is an indicator of being in the room.

We therefore add another attribute to indicate whether or not an HCP badge is imputed to be in a patient room. This attribute turns positive upon contact with a patient room’s interior anchor and becomes negative when the HCP badge subsequently contacts some other anchor outside the patient room. Each history entry is enhanced so that ${\mathcal{H}}[t][a]$ (history at time *t* for HCP *a*) has an attribute *a*. *inroom* indicating whether or not *a* is inferred to be in a patient room; if applicable, another attribute *a*. *room* specifies the room number. We include the *room* and *inroom* attributes in the imputed history files, which accompany this article.

### Study Limitations

The IRB agreement does not allow the identification of HCPs, nor does the study have patient data, patient outcomes, or infection tracing. For HCPs working in multiple shifts, badges worn could be different in each shift, which could limit the kinds of questions in, e.g., social network analysis, where graph nodes ideally represent persons rather than job roles or badges. A further complication is the protocol we used for badge distribution at the start of each shift: to minimize the delay imposed on HCPs, we turned on badges just before the shift (also checking batteries and clearing badge memory) so that an HCP could quickly pick up and wear a badge. Some of these available badges were not picked up; they were powered off within half an hour of the shift start. Consequently, extra badge identifiers may appear in the data, though not in the interior of the MICU (patient rooms, work areas, nurse stations).

Members of our team were not always present in the MICU: we cannot know if an HCP removed a badge, left the MICU, or other scenarios. As noted previously in this section, although UWB distance estimation has excellent accuracy, results for contact measurement are further approximated due to the timing of sensing proximity. Many research questions one might hope to investigate are outside the scope of the data: finer spatial resolution (e.g., more anchors, reduced threshold for contact distance), more job types, more days of observation, and so forth.

## Data Records

The dataset is available at the Figshare database^41^ (10.6084/m9.figshare.28826414.v3). Data are in three parts under data folder in the repository: raw data (fulldata.xz), contact intervals (contact_intervals/intervalsXX.json.xz) and imputed histories (histories/historiesXX.json.xz) with XX being a 2-digit index for each shift; there is also a spreadsheet (badgelocations.xlsx) with notes on anchor locations during the study, and a placement005.yaml file which has coordinates and badge labels, in the supp folder. An image of the MICU layout is in figures/iculayout.png; the coordinates in placement005.yaml specify, in terms of pixels of this image, where anchors are located. The raw data is stored in a single compressed file containing all shifts, while contact intervals and histories are stored in separate compressed files for each shift.

Provenance of the data follows from the chain of data acquisition: collection proceeds from badge memory to a smartphone collection app, then to the vendor’s cloud database, and finally from the vendor’s database to our own repository via API calls to the Instant-Trace system.

The complete raw data file spans nearly one week of day and night shifts. The raw data contains approximately 15 million records. The program that invoked the Instant-Trace API to retrieve data copied all record fields except for the three-byte badge identifiers and a download timestamp. This program changed only badge detection times, as these were UTC timestamps, by subtracting five hours to get local time. Conversion of raw data to contact intervals considered 16 shifts, numbered 0–15. However, the contact interval data files are for shifts 1–14, and shift 0 contains a walk-through described in the Technical Validation section. Odd-numbered shifts are night shifts, and even-numbered shifts are day shifts. Badge distribution to HCPs started late on shift 1, so it has fewer contacts than other night shifts. It was during shift 15 that we removed anchors and halted the study.

All the data files use JSON encoding. The same format applies to both raw data and contact interval files. Table 1 shows the format of one example recording of an observation of contact between badges pr014 and pr037. Field (c) in the table is a string representation of a Python datetime object; such Python objects are convenient for calculations, extracting time of day, and so forth. Field (d) contains a value for a sensed distance; see the Methods Section on how we used this value. Table 2 shows a fragment from the imputed history, which has a more complex data structure: for each second of history, there is a mapping that is inspired by an edge-adjacency list of a contact graph. Our data files are compressed using the Lempel-Ziv-Markov chain algorithm from the standard Python library, lzma; for example, generated imputed history data is 4.5GB uncompressed and 13MB compressed. The compressed form can be directly read by Python programs.

**Table 1:** Format of Raw Data and Contact Interval Records.

	["pr014”, "pr037”, "datetime(2023,4,25,7,39,43)”, 74, 0]
(a)	pr014 is the reporting badge label
(b)	pr037 is the detected badge label (other end of contact)
(c)	datetime(2023,4,25,7,39,43) is the start time of contact; for raw data, even end-of-interval records have the start time
(d)	74 is the distance in inches (see Methods Section)
(e)	0 is the duration of contact; in raw data, zero means start, nonzero means end of contact interval

The same format is used for both raw data and contact intervals, the main difference being (e) the duration field. For raw data, zero duration indicates the start of contact, whereas in the contact intervals, this field will never be zero: (e) in each contact interval will be the length, in seconds, of the estimated interval of contact.

**Table 2:** Format of a Fragment of Imputed History.

"datetime(2023,4,21,7,2,57)”: {
"n004”:{"contacts”:["pr023”,"ss004”,"pr010”,"n046”,"pr043”],"state”:null},
"ss017”:{"contacts”:["b097”,"n009”],"state”:"room”:7,"inroom”:true},
...

This is part of one history entry for 21 April, 2023, at 07:02:57. Our code represents history as a dictionary indexed by a datetime object, a standard Python class. Each indexed item is itself a dictionary, indexed by HCP badge identifier. For this particular datetime, nurse badge n004 has five concurrent contacts with other HCP badges; support staff badge ss017 has contact with nurse badge n009, anchor b097, and is in patient room 7. If H is a history containing this fragment, then H[datetime(2023,4,21,7,2,57)]["ss017”]["contacts”] is ["b097”,"n009”].

## Technical Validation

We validated the data in three ways: low-level, intermediate-level, and high-level. Low-level validation consisted of manual tests using several badges by two people and a measuring tape fixed on the floor. Badges can be configured to sound an alarm (an annunciator feature) and flashlights when contact is detected. By repeatedly setting different contact distances on the Instant-Trace dashboard, uploading the distance to the badges, and experimenting with bringing the badges within the specified distance, we were able to confirm the vendor’s claim that measurements are precise within a few inches (10 cm). The intermediate-level validation took place the day before the deployment after all anchors had been placed within the MICU. Two people, each wearing two badges, visited each instrumented room in the MICU; each visit to a room was noted on a smartphone memo app. After scanning the badges to upload data, we were able to reconstruct the trajectory from API-acquired contact records with no errors observed.

**Fig. 4:**
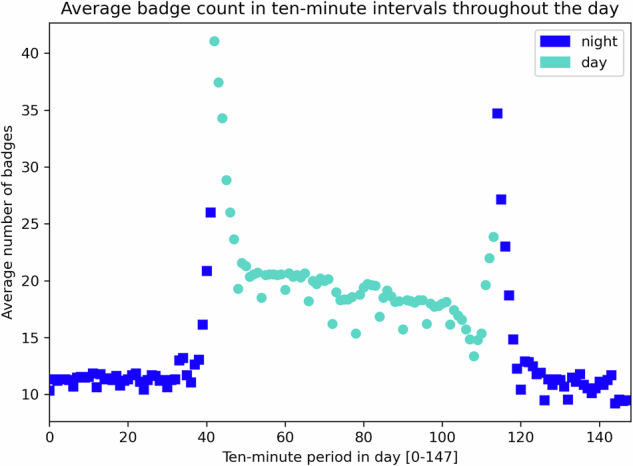
Mean number (*y*-axis) of HCP badges detected for each 10-minute period in a day (*x*-axis), over shifts 1-14; the criterion for inclusion in the badge count is having at least one contact with another badge (HCP or anchor) during the period. Blue square markers are night shift points; green circle markers are for day shift.

Although low-level and intermediate-level validation convince us that the hardware and data extraction processes are trustworthy, there remains the consideration of high-level validity. Starting from the roster sizes, where we know there are more HCPs in a day shift than a night shift, the question is whether or not this fact can be confirmed in the study data. Figure 4 shows the mean badge count, taken over shifts 1-14, for each ten-minute interval of a day (148 = 24*6 intervals). Input for the mean count was obtained from the imputed history files by looking for any badge that had at least one contact (HCP or anchor) during a specified ten-minute interval *h*, for *h* ∈ [0, 147]. Day shifts begin at 07:00 and end at 19:00. The peaks seen in the figure correspond to shift changes and are due to three factors: (*i*) HCPs can arrive early or leave later than the shift change; (*ii*) the handout protocol, described in the Study Limitations subsection, temporarily introduces some extra badges to the count; (*iii*) badges turned in may not be immediately scanned for data and turned off (powering off a badge off loses its data); during this period awaiting scanning, a badge could have a contact, say with a nearby anchor, and be counted in the first hour of the next shift. The result of these effects is an interval in the imputed history where badges from two consecutive shifts are detected, so the observed badge count is larger than that of either shift during this interval. If we ignore the peaks, mean badge counts corroborate that day shifts have more HCPs than night shifts.

## Usage Notes

Our data and code are freely accessible from the Figshare repository without restrictions or review requirements. Under code folder, the script make_intervals.py reads the raw data and generates contact intervals for each shift, while make_histories.py imputes the histories based on these intervals. The module validation.py supports basic validation by visualizing the average number of HCP badges throughout the day. The Jupyter notebook IowaMICU2023.ipynb illustrates how to count badges per shift using processed contact interval files. Additional scripts such as showbadge.py and extractspread.py enable spatial visualization of badge placements and extraction of anchor metadata, respectively. All the provided code can be executed by calling to python command with the script file path from the root directory of the repository.

## Data Availability

The code used to read and process the raw data — producing both intermediate formats and final results (described in the Methods and Technical Validation sections) — is available in the code folder within the same Figshare repository as our dataset^41^. The Python code is open-source and relies solely on standard, publicly available libraries commonly used in desktop or laptop environments. It is designed to be self-contained and executable without requiring any specific setup beyond a standard Python environment.
